# Postural Control During Single-Leg Stance Under Degraded and Occluded Visual Conditions in Healthy Young Adults

**DOI:** 10.3390/jfmk11020205

**Published:** 2026-05-23

**Authors:** Anna Chalkia, Georgios Tsigaras, Alexandra Kallistratou, Paris Iakovidis, Dimitrios Lytras, Christoforos Pando, Ilias Kallistratos

**Affiliations:** 1 Laboratory of Basic and Applied Research in Physiotherapeutic Rehabilitation, Department of Physiotherapy, Faculty of Health Sciences, International Hellenic University, 57400 Thessaloniki, Greece; axalkia@ihu.gr (A.C.); tsigarasg@ihu.gr (G.T.); alexkallgi@gmail.com (A.K.); xpando44@gmail.com (C.P.); elikall@ihu.gr (I.K.); 2 Laboratory of Biomechanics & Ergonomics, Department of Physiotherapy, Faculty of Health Sciences, International Hellenic University, 57400 Thessaloniki, Greece; piakov@ihu.gr

**Keywords:** postural control, sensory reweighting, visual occlusion, low illumination, single-leg stance, center of pressure

## Abstract

**Background:** Vision is a key sensory system for postural regulation; however, the effects of degraded visual input and complete visual occlusion on static balance are not fully understood. The aim of the present study was to compare postural control during single-leg stance under two reduced-vision conditions (eyes open in darkness vs. complete visual occlusion) in healthy young adults and examine the potential influence of sex and mild visual deficits. **Materials and Methods:** This within-subject laboratory study included 42 healthy young adults (21 males, 21 females; mean age 20.67 ± 0.48 years). Participants performed three valid 20 s single-leg stance trials on a force platform under two visual conditions: eyes open in darkness and complete visual occlusion using an opaque mask. The order of conditions was randomized and counterbalanced, and the mean value of the three valid trials under each condition was used for analysis. Postural sway outcome variables included CoP Area, Oscillation Width, Oscillation Height, Total Displacement, and Mean Velocity. A two-way mixed-design ANOVA examined the effects of visual condition and sex. Additional mixed ANCOVA analyses were performed using body weight as a covariate to verify whether the sex-related findings remained after adjustment for body weight. Exploratory subgroup analyses based on mild visual deficits were performed using independent-samples *t*-tests. **Results:** No significant overall main effect of visual condition was observed for any postural sway variable (all *p* > 0.05). However, a significant condition × sex interaction was found for CoP Area (*F*(1,40) = 9.910, *p* = 0.003, *η*^2^p = 0.199), indicating different response patterns between males and females across conditions. Significant main effects of sex were also found for Total Displacement (*F*(1,40) = 9.212, *p* = 0.004, *η*^2^p = 0.187) and Mean Velocity (*F*(1,40) = 9.090, *p* = 0.004, *η*^2^p = 0.185), with males showing higher values overall. The sex-related findings for CoP Area, Total Displacement, and Mean Velocity remained significant after adjustment for body weight. No significant sex effects were found for Oscillation Width or Oscillation Height, and no significant differences were observed between participants with and without mild visual deficits in either condition (all *p* > 0.05). **Conclusions:** Altered visual input did not produce a uniform overall effect on postural sway during single-leg stance in healthy young adults. Instead, the findings indicate a more differentiated pattern, with a sex-specific response for CoP Area and overall sex-related differences in Total Displacement and Mean Velocity that were not explained by body weight. Mild visual deficits were not associated with significant balance alterations under the present experimental conditions. These findings support a more nuanced interpretation of postural regulation under reduced visual input and highlight the importance of considering individual characteristics, particularly sex, in balance assessment.

## 1. Introduction

Postural control is a complex sensorimotor function that depends on the continuous integration of visual, proprioceptive, and vestibular information in order to maintain orientation and stability under changing environmental and task demands [1,2,3]. Rather than relying simply on the presence or absence of sensory input, the central nervous system dynamically adjusts the relative contribution of the available sensory channels according to the sensory context. When one source of information becomes degraded, ambiguous, or unavailable, postural regulation may adapt by increasing reliance on other sensory inputs, a process commonly described as sensory reweighting [1,2,4].

Vision plays a particularly important role in postural orientation and stabilization during upright stance, with its contribution becoming more pronounced under demanding conditions such as single-leg stance [1,2,5,6]. However, the contribution of vision to balance is not uniform across sensory contexts [1,2]. In many experimental paradigms, visual input is manipulated dichotomously, most commonly by comparing eyes-open and eyes-closed conditions. Although this approach has yielded important insight into the contribution of vision to postural control, it may overlook a relevant distinction between the complete absence of visual input and situations in which the eyes remain open but the available visual information is functionally uninformative or unreliable [2,7].

This distinction may be especially relevant in environments with extremely low illumination. Standing with the eyes open in darkness may not be functionally equivalent to complete visual occlusion or eye closure, as eyes-open and eyes-closed conditions appear to represent distinct functional brain states even in the absence of meaningful visual stimulation [7,8]. In the former condition, the visual system may remain operational despite the absence of useful external visual references, potentially exposing the postural control system to limited or degraded visual information. In the latter condition, visual input is fully blocked, which may promote a more direct reliance on non-visual sensory inputs, including proprioceptive and vestibular information [2,4]. From this perspective, the key issue is not only whether visual input is present but whether it is sufficiently informative to support postural stabilization.

From an applied perspective, this distinction is relevant because visual input manipulations are frequently incorporated into balance assessment, rehabilitation, and athletic training protocols [9,10,11]. However, “darkness” and “visual occlusion” may impose different sensory demands. Training or testing with the eyes open in darkness may challenge the postural control system through the availability of minimal, unreliable, or ambiguous visual information, whereas complete visual occlusion removes visual input more definitively and may therefore promote greater reliance on non-visual sensory cues [12]. Clarifying whether these two conditions elicit different postural responses is important for the interpretation of balance assessments and the selection of sensory-challenging strategies in clinical and training settings.

Single-leg stance provides a particularly relevant postural task for investigating this question because it imposes greater postural demands than bipedal stance and may therefore be more sensitive to subtle changes in sensory organization and stabilization strategy under reduced-vision conditions [1]. In addition to the visual condition itself, postural behavior may also be influenced by individual factors that shape stabilization strategies. Among these, sex may represent a relevant modifying factor. Although sex-related differences in postural sway have been reported in the literature, findings remain inconsistent and appear to depend on the characteristics of the population studied as well as the specific postural task employed. Recent evidence suggests that such differences may be limited in young adults under less demanding conditions, but may become more apparent under more challenging balance tasks or older populations [13,14]. Accordingly, sex was included as a relevant modifying factor in the present study.

Visual status represents another potentially relevant modifying factor. Vision plays a primary role in postural control by providing information about body orientation in space, and its absence or degradation has been associated with increased postural sway and altered balance strategies [15]. Individuals with reduced or impaired visual function may rely differently on non-visual sensory inputs, particularly when visual information becomes degraded or unavailable. Accordingly, even subtle variations in visual function—although not necessarily sufficient to warrant exclusion—may influence responses under low-light or visually ambiguous conditions and therefore warrant exploratory consideration in studies of reduced-vision postural control.

To minimize age-related heterogeneity in postural performance, the present study focused on healthy young adults. Young adulthood provides a relatively homogeneous period for examining postural control, with fewer age-related confounding influences than those observed during developmental stages or later adulthood, when balance performance and sensory integration may show greater variability [13,16,17]. Examining the study question in a young adult sample therefore allows a more controlled evaluation of postural behavior under experimentally reduced visual conditions.

Despite the extensive literature on visual contributions to balance, limited evidence has directly compared eyes-open standing in darkness with complete visual occlusion during single-leg stance within the same participants. Moreover, whether individual characteristics such as sex and mild visual deficits may modify responses to these two reduced-vision conditions remains insufficiently clarified.

Therefore, the primary aim of the present within-subject laboratory study was to compare single-leg static balance under two forms of reduced visual input: (a) eyes open in darkness and (b) complete visual occlusion using an opaque eye mask. Secondary aims were to examine whether postural responses under these conditions differed according to sex and mild visual deficits. By directly comparing these two reduced-vision conditions within the same participants, the study sought to determine whether they should be interpreted as functionally equivalent sensory challenges or distinct contexts of postural regulation. We hypothesized that the two visual conditions would not be functionally equivalent and that postural responses under complete visual occlusion might differ according to sex and mild visual deficits.

## 2. Materials and Methods

### 2.1. Study Design

This within-subject laboratory study was conducted at the Department of Physiotherapy, International Hellenic University, Thessaloniki, Greece, in the Laboratory of Basic and Applied Research in Physiotherapeutic Rehabilitation. The study protocol was approved by the institutional bioethics committee of the university (approval no. EC-10/2025). All procedures were conducted in accordance with the principles of the Declaration of Helsinki, and written informed consent was obtained from all participants prior to data collection.

### 2.2. Participants

Forty-two healthy, young adults were recruited for this study. This age group was intentionally selected to reduce age-related heterogeneity in postural performance and examine the study question in a population less likely to exhibit the age-related changes in postural control reported in later adulthood [13,16]. Eligibility was established through standardized screening based on medical history and predefined criteria.

Inclusion criteria were: (1) age between 18 and 25 years and (2) absence of known neurological or vestibular disorders.

Exclusion criteria were: (1) severe uncorrected visual impairment, (2) any self-reported history of dizziness or vestibular dysfunction, and (3) current musculoskeletal conditions, including recent or recurrent lower-limb injury or ankle dysfunction, that could influence single-leg stance performance.

A brief preliminary questionnaire was administered to record the presence of mild visual deficits, based on participants’ reported visual history and corrective lens prescription where applicable.

### 2.3. Measurements

#### Postural Sway Assessment

Postural sway was assessed using a portable force platform (K-Force Plates, model MP.0703, Kinvent^®^, Montpellier, France), with a sampling frequency of 250 Hz. Prior to each measurement session, the force plates were zeroed and calibrated according to the manufacturer’s specifications to ensure measurement accuracy. The Center of Pressure (CoP) trajectory was analyzed in the horizontal (mediolateral and anteroposterior) plane, representing postural sway during single-leg stance.

The outcome variables derived from the force-platform recordings included CoP Area (mm^2^), representing the total sway area covered during the trial; Oscillation Width (mm), corresponding to mediolateral CoP displacement; and Oscillation Height (mm), corresponding to anteroposterior CoP displacement. In addition, Total Displacement (mm), representing the total length of the CoP trajectory during the trial, and Mean Velocity (mm/s), representing the average speed of CoP displacement, were also recorded. These variables were used as indicators of postural control, with higher values reflecting greater postural sway and reduced stability.

### 2.4. Experimental Protocols

**Figure 1:**
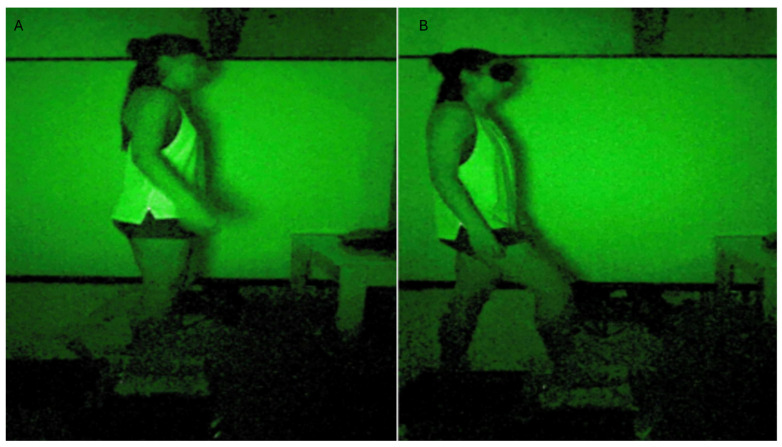
Representative single-leg stance assessment under the two experimental visual conditions: (**A**) eyes open in a dark room (<1 lux) and (**B**) complete visual occlusion using an opaque eye mask. The assessment was performed on the dominant lower limb on a force platform under infrared/night vision supervision.

The balance assessment protocol was completed during a single testing session and is illustrated in Figure 1. Participants were evaluated under two visual conditions: (a) eyes open in darkness and (b) complete visual occlusion in the same setting using an opaque eye mask. All assessments were conducted under very low ambient illumination (<1 lux), which was verified prior to testing using a digital lux meter. To minimize residual visual cues, the testing room was free of illuminated displays, external light sources, reflective surfaces, and high-contrast spatial references. Participant safety was ensured through continuous supervision using a night vision camera equipped with infrared LEDs (model ZC-M6, Shenzhen Tianzhishan Technology Co., Ltd., Shenzhen, China) that were not visible to the participants. In the eyes-open-in-darkness condition, participants remained in the darkened environment for a standardized dark-adaptation period of 5 min before the beginning of data collection in order to reduce the transient effects of visual adjustment to low illumination. This condition was intended to represent a state of minimal and potentially unreliable visual input rather than complete visual deprivation. In the visual occlusion condition, participants wore an opaque eye mask to ensure complete elimination of visual input.

The dominant lower limb was defined as the preferred leg used to kick a ball. Participants were instructed to perform a 20 s single-leg stance on the dominant lower limb while maintaining the test position as steadily as possible. During testing, the arms were kept alongside the trunk, and the non-supporting limb remained flexed without contacting either the stance limb or the force platform. Before formal data collection, one familiarization trial was performed to ensure that participants understood the testing procedure and were accustomed to the experimental setting.

To minimize potential order effects, the sequence of the two visual conditions was randomly assigned for each participant using the Research Randomizer software (version 4) [18]. Thus, some participants performed the eyes-open-in-darkness condition first, whereas others began with the visual occlusion condition. For each visual condition, three valid trials were recorded. A trial was considered valid if the participant maintained the required single-leg stance for the full 20 s period without loss of balance, contralateral foot contact with the floor, contact of the non-supporting limb with the stance limb, or the need for external assistance. Trials that did not meet these criteria were discarded and repeated. A standardized rest interval of 30 s was provided between consecutive trials, while a 60 s rest interval was allowed between conditions in order to minimize fatigue and transient instability. The mean value of the three valid trials obtained under each condition was used for statistical analysis in order to improve measurement reliability and reduce trial-to-trial variability.

### 2.5. Sample Size Estimation

An a priori sample size estimation was performed using G*Power software (version 3.1.9.7) for a mixed-design ANOVA (repeated measures, within–between interaction), based on the primary analytical model of the study. In the absence of directly comparable previous studies using the same reduced-vision conditions and single-leg stance protocol, a medium effect size was assumed (f = 0.25), according to Cohen’s conventions [19]. With an alpha level of 0.05, statistical power of 0.85, two groups, and two repeated measurements, the required total sample size was estimated at 38 participants. Therefore, the final sample of 42 participants was considered adequate for the planned analyses.

### 2.6. Statistical Analysis

All statistical analyses were performed using IBM SPSS Statistics (version 25.0; IBM Corp., Armonk, NY, USA). Descriptive statistics are presented as mean ± standard deviation (SD). For each participant, the mean value of the three valid trials obtained under each visual condition was calculated and used for all subsequent analyses. Statistical significance was set at α = 0.05. The normality of the data distribution was assessed using the Shapiro–Wilk test and Q–Q plots. Separate two-way mixed-design analyses of variance (mixed ANOVA) were conducted for each postural sway outcome variable, including CoP Area, Oscillation Width, Oscillation Height, Total Displacement, and Mean Velocity. In each model, visual condition (eyes open in darkness vs. complete visual occlusion) was treated as a within-subject factor, while sex (male vs. female) was included as a between-subject factor. Main effects of condition and sex, as well as the interaction effect (condition × sex), were evaluated. Effect sizes were reported using partial eta squared (*η*^2^p). Where significant interaction effects were identified, Bonferroni-adjusted pairwise comparisons were performed as appropriate.

Additional mixed ANCOVA analyses were performed for each postural sway outcome to examine whether the observed sex-related findings remained after adjustment for body weight. In these models, visual condition was treated as the within-subject factor, sex as the between-subject factor, and body weight as the covariate.

Subgroup analyses based on mild visual deficits were performed in an exploratory manner. Differences between subgroups were examined within each visual condition using independent samples *t*-tests.

## 3. Results

The descriptive characteristics of the study sample are presented in Table 1. A total of 42 healthy young adults participated in the study. The mean age of the participants was 20.67 ± 0.48 years. The sample was equally distributed by sex, comprising 21 males (50%) and 21 females (50%). Most participants exhibited right lower limb dominance (90.5%), while a smaller proportion reported left dominance (9.5%). Regarding visual status, 40.5% of the participants were classified as having mild visual deficits, whereas 59.5% reported no such deficits. No missing data were observed for the analyzed variables.

### 3.1. Postural Sway Variables by Sex

A two-way mixed-design ANOVA was performed to assess the effects of visual condition and sex on postural sway variables during single-leg stance. For CoP Area, no significant main effect of visual condition was observed (*F*(1,40) = 0.014, *p* = 0.906, *η*^2^p = 0.000), indicating that CoP Area did not differ overall between the eyes-open-in-darkness and visual occlusion conditions. Likewise, no significant main effect of sex was found (*F*(1,40) = 1.634, *p* = 0.208, *η*^2^p = 0.039). However, a statistically significant condition × sex interaction was identified (*F*(1,40) = 9.910, *p* = 0.003, *η*^2^p = 0.199), indicating different response patterns between sexes across visual conditions. Descriptively, males exhibited higher CoP Area values under complete visual occlusion than under the eyes-open-in-darkness condition, whereas females demonstrated the opposite directional pattern (Table 2). Specifically, CoP Area increased by approximately 21.6% in males under visual occlusion compared with the eyes-open-in-darkness condition, whereas in females, CoP Area was approximately 28.0% higher in the eyes-open-in-darkness condition than under visual occlusion.

For Total Displacement, no significant main effect of visual condition was observed (*F*(1,40) = 0.446, *p* = 0.508, *η*^2^p = 0.011), and the condition × sex interaction was also not significant (*F*(1,40) = 0.406, *p* = 0.528, *η*^2^p = 0.010). However, a significant main effect of sex was found (*F*(1,40) = 9.212, *p* = 0.004, *η*^2^p = 0.187), indicating that males demonstrated greater Total Displacement overall compared with females, irrespective of visual condition. Quantitatively, Total Displacement was approximately 20.9% higher in males than in females in the eyes-open-in-darkness condition and 26.8% higher under visual occlusion.

Similarly, for Mean Velocity, no significant main effect of visual condition was found (*F*(1,40) = 0.411, *p* = 0.525, *η*^2^p = 0.010), and no significant condition × sex interaction was observed (*F*(1,40) = 0.481, *p* = 0.492, *η*^2^p = 0.012). In contrast, a significant main effect of sex emerged (*F*(1,40) = 9.090, *p* = 0.004, *η*^2^p = 0.185), showing that males had higher Mean Velocity values overall than females. Quantitatively, Mean Velocity was approximately 20.5% higher in males than in females in the eyes-open-in-darkness condition and 26.9% higher under visual occlusion.

No statistically significant main effects of visual condition, condition × sex interactions, or main effects of sex were identified for Oscillation Width or Oscillation Height (all *p* > 0.05) (Table 2). Specifically, for Oscillation Width, the main effect of condition was *F*(1,40) = 1.373, *p* = 0.248, *η*^2^p = 0.033; the interaction effect was *F*(1,40) = 0.585, *p* = 0.449, *η*^2^p = 0.014; and the main effect of sex was *F*(1,40) = 1.197, *p* = 0.280, *η*^2^p = 0.029. For Oscillation Height, the main effect of condition was *F*(1,40) = 0.000, *p* = 0.993, *η*^2^p = 0.000; the interaction effect was *F*(1,40) = 1.091, *p* = 0.303, *η*^2^p = 0.027; and the main effect of sex was *F*(1,40) = 1.348, *p* = 0.253, *η*^2^p = 0.033.

### 3.2. Body Weight-Adjusted ANCOVA Analyses

Additional mixed ANCOVA analyses were conducted to examine whether body weight influenced the observed sex-related findings. For each postural sway outcome, visual condition was entered as the within-subject factor, sex as the between-subject factor, and body weight as the covariate.

Overall, body weight was not a significant covariate for any of the examined postural sway outcomes. For CoP Area, the condition × sex interaction remained statistically significant after adjustment for body weight (*F*(1,39) = 5.856, *p* = 0.020, *η*^2^p = 0.131), whereas body weight was not significant (*F*(1,39) = 0.111, *p* = 0.741, *η*^2^p = 0.003).

For Total Displacement, the main effect of sex remained statistically significant after adjustment for body weight (*F*(1,39) = 4.751, *p* = 0.035, *η*^2^p = 0.109), whereas body weight was not significant (*F*(1,39) = 0.001, *p* = 0.979, *η*^2^p = 0.000). Similarly, for Mean Velocity, the main effect of sex remained statistically significant after adjustment for body weight (*F*(1,39) = 4.705, *p* = 0.036, *η*^2^p = 0.108), whereas body weight was not significant (*F*(1,39) = 0.000, *p* = 0.984, *η*^2^p = 0.000).

For Oscillation Width and Oscillation Height, no significant main effects of sex or condition × sex interactions were observed after adjustment for body weight. Body weight was also not a significant covariate for Oscillation Width (*F*(1,39) = 0.388, *p* = 0.537, *η*^2^p = 0.010) or Oscillation Height (*F*(1,39) = 0.126, *p* = 0.724, *η*^2^p = 0.003).

These findings indicate that the significant sex-related findings observed for CoP Area, Total Displacement, and Mean Velocity were not explained by body weight.

### 3.3. Postural Sway Variables by Mild Visual Deficits

Independent-samples *t*-tests were conducted to examine whether postural sway differed between participants with and without mild visual deficits under each visual condition (Table 3). No statistically significant differences were observed between groups for any of the postural sway variables in either condition (all *p* > 0.05). Specifically, CoP Area did not differ significantly between participants with and without mild visual deficits in the eyes-open condition (*t*(40) = 1.037, *p* = 0.306) or the visual occlusion condition (*t*(40) = 0.516, *p* = 0.608). Similarly, no significant between-group differences were found for Total Displacement, Mean Velocity, Oscillation Width, or Oscillation Height in either visual condition (all *p* > 0.05). Descriptively, participants with mild visual deficits showed somewhat higher values for several variables under the eyes-open condition; however, these differences did not reach statistical significance and should therefore be interpreted cautiously. The largest between-group difference was observed for Oscillation Height in the eyes-open condition; however, this effect did not reach statistical significance (*t*(40) = 1.781, *p* = 0.083).

## 4. Discussion

The present study aimed to compare postural control during single-leg stance under two forms of reduced visual input, namely eyes open in darkness and complete visual occlusion, while also examining the potential role of sex and mild visual deficits. Contrary to the initial expectation that reduced visual reliability might exert a uniform destabilizing effect, the results did not demonstrate a significant overall main effect of visual condition on any of the examined postural sway variables. Instead, the most notable finding was a significant condition × sex interaction for CoP Area, indicating that males and females responded differently to the two visual conditions. Specifically, males exhibited higher CoP Area values under complete visual occlusion than under the eyes-open-in-darkness condition, whereas females showed the opposite descriptive pattern, with higher CoP Area values under the eyes-open condition. In addition, males demonstrated significantly greater Total Displacement and Mean Velocity overall, irrespective of visual condition. These sex-related findings remained significant after adjustment for body weight, whereas no significant differences were observed according to mild visual deficits. Taken together, these findings suggest that the postural response to altered visual input in healthy young adults is not characterized by a simple global effect of vision reduction but rather by a more nuanced pattern in which sex-related factors appear to modulate specific aspects of postural sway.

These findings can still be interpreted within the framework of sensory reweighting. Sensory reweighting refers to the dynamic process by which the central nervous system adjusts the relative contribution of visual, vestibular, and proprioceptive information according to the reliability of each source in a given context [2,4,20,21]. In the present study, the absence of a significant overall condition effect suggests that, at least at the group level, healthy young adults were able to maintain comparable postural performance under both degraded and absent visual input. This may reflect the efficiency and flexibility of sensory integration mechanisms in young adulthood, a period generally associated with mature sensory processing. Rather than demonstrating a universal destabilizing effect of one condition over the other, the current data suggest that participants as a whole were capable of adapting their postural strategy sufficiently to preserve stability across both sensory contexts. Importantly, the absence of a significant overall condition effect should not be interpreted as evidence that visual manipulation was irrelevant. Rather, it may indicate that healthy young adults were able to compensate effectively under both reduced-vision conditions, thereby maintaining comparable overall postural performance.

The absence of a uniform condition effect therefore does not necessarily reduce the relevance of the research question. Instead, it suggests that the distinction between degraded visual input and complete visual occlusion may be better understood as a context-dependent sensory challenge rather than as a simple hierarchy of visual deprivation [2,4]. This interpretation is particularly relevant for healthy young adults, in whom efficient sensory compensation may mask group-level differences while still allowing subgroup- or variable-specific effects to emerge [2,4].

At the same time, the significant interaction observed for CoP Area indicates that this adaptive process may not be expressed identically across sexes. This is an important point because it shifts the interpretation away from a purely condition-centered explanation toward a more interactive one. One plausible interpretation is that males and females may differ in the way they adapt postural control under altered visual conditions, even when their overall level of stability remains broadly comparable. The opposite directional pattern observed in CoP Area suggests that the two sexes may adopt somewhat different stabilization strategies when visual information is degraded versus fully removed. In males, complete visual occlusion may have required greater reliance on non-visual sensory systems and may have been associated with a larger sway area. In females, by contrast, the eyes-open-in-darkness condition may have imposed a greater challenge, possibly because nominally available but unreliable visual information introduced ambiguity into sensory integration, consistent with evidence that postural regulation depends on the reliability of available sensory information rather than simply its presence or absence [2,21]. Although these interpretations remain cautious and inferential, they are conceptually consistent with the broader literature showing that multisensory contributions to postural control are dynamically reweighted according to contextual demands [4,20] and that environmental context can meaningfully shape postural behavior [22]. This interpretation is also broadly compatible with the literature suggesting that postural responses may vary according to sex, particularly as a function of task and sensory condition [13,14]. Potential moderators of these responses may include anthropometric characteristics, sensory integration processes, and neuromotor control strategies.

The present findings also contribute to the broader discussion regarding the functional distinction between degraded vision and complete visual absence. Previous work has suggested that altered or intermittently disrupted visual input may place substantial demands on sensorimotor processing because the nervous system may continue to allocate processing resources to visual information that is nominally available but functionally limited [23,24]. However, the current results indicate that such an effect does not necessarily emerge as a robust overall group phenomenon in healthy young adults performing a static single-leg stance task. Instead, any destabilizing influence of altered visual input appears to be selective and dependent on the interaction with participant characteristics, particularly sex. In this sense, the present study refines rather than contradicts the sensory reweighting model: it suggests that the effects of degraded versus absent vision may be real, but they are not uniform across all sway variables or all individuals. This is compatible with recent evidence indicating that sensory reweighting is not a fixed response but a dynamic and context-sensitive process, influenced by task demands, sensory context, and individual characteristics [4,25,26].

The significant sex-related findings for Total Displacement and Mean Velocity further support the view that sex should be considered as a relevant factor in studies of postural regulation. In the present sample, males demonstrated higher values for both variables across conditions, indicating greater overall sway excursion and higher sway velocity. Since greater CoP excursion and higher sway velocity are commonly interpreted as indicators of reduced postural steadiness [27,28], these findings suggest that males, on average, exhibited a less stable postural profile than females during the single-leg stance task. Importantly, this difference was not condition-specific, as no significant interaction with visual condition was observed for either variable. Thus, the sex effect appears to reflect a more general feature of postural control rather than a differential sensitivity to altered visual input for these measures.

Several mechanisms may account for these sex-related differences. One possibility is that males and females differ in biomechanical characteristics and postural control strategies, both of which may influence sway magnitude and velocity during single-leg stance [13,29]. Anthropometric characteristics have been shown to affect stabilometric measures, particularly those related to CoP displacement and sway behavior during upright stance [29]. More specifically, variables such as body height, body mass, foot dimensions, lower-limb alignment, and differences in center of mass position may influence CoP displacement and sway velocity. In the present sample, males had significantly greater body height and body weight than females, whereas BMI did not differ significantly between groups. Therefore, the observed sex-related differences in Total Displacement and Mean Velocity may partly reflect body size-related biomechanical factors rather than sex-related differences in neurosensory control alone. Accordingly, the observed sex-related differences should not be interpreted solely as reflecting differences in neurosensory control strategies but may also reflect biomechanical or anthropometric influences. To further examine the potential influence of body weight, additional mixed ANCOVA analyses were performed using body weight as a covariate. These analyses showed that body weight was not a significant covariate for any of the examined postural sway outcomes. Importantly, the condition × sex interaction for CoP Area and the main effects of sex for Total Displacement and Mean Velocity remained statistically significant after adjustment for body weight. Therefore, the significant sex-related findings observed in the present study were not explained by body weight, although broader anthropometric and biomechanical characteristics may still contribute to the organization of postural corrections. At the same time, the absence of a significant main effect of sex for CoP Area suggests that sex-related differences may not influence all sway variables equally, which is consistent with evidence indicating that the interpretation of postural sway depends on the specific outcome measure considered [13,27]. This again highlights the importance of treating postural sway as a multidimensional construct rather than assuming that all CoP-derived variables represent the same control mechanism [27,28].

The lack of significant sex effects for Oscillation Width and Oscillation Height is also informative. These findings indicate that sex-related differences were not generalized across all planes or all spatial descriptors of sway. Instead, they were more clearly expressed in variables reflecting the overall length and dynamic activity of the sway trajectory [27,28]. This pattern may imply that males and females differed more in the amount and velocity of corrective behavior than in the absolute boundaries of mediolateral or anteroposterior sway. In other words, the sexes may have occupied similar sway ranges but may have relied on somewhat different control strategies to remain stable. Such an interpretation is broadly consistent with the idea that postural control depends on the interaction between task demands, outcome measures, and underlying control processes, rather than being fully captured by a single global sway metric [13,27,28].

No significant differences were observed between participants with and without mild visual deficits under either visual condition. This suggests that mild visual deficits, as captured in the present study, were not sufficient to produce measurable differences in postural control during single-leg stance in this sample of healthy young adults. This finding is plausible, given that the mild visual deficits were not clinically quantified and likely represented relatively mild or well-compensated visual limitations. In such cases, the central nervous system may still be able to maintain effective postural regulation through flexible multisensory compensation [2,4]. Moreover, because the participants were young adults, the absence of measurable subgroup differences may also be consistent with evidence suggesting that healthy young adults can maintain relatively stable postural performance under altered visual conditions, particularly when task demands remain controlled or only moderately challenging [30]. Although some descriptively higher sway values were observed in the mild visual deficits subgroup under the eyes-open condition, these did not reach statistical significance. The largest non-significant between-group difference was observed for Oscillation Height in the eyes-open condition, which may indicate a possible tendency worthy of future exploration, but it cannot be interpreted as evidence of a reliable subgroup effect in the present dataset.

These findings have several implications for the interpretation of altered visual input in balance assessment and training. First, they suggest that reduced vision should not automatically be assumed to destabilize all individuals in the same way. The present results indicate that healthy young adults can maintain similar overall postural performance across degraded and absent visual conditions, particularly when task demands remain controlled and no additional sensory or mechanical challenge is introduced [30]. Second, the fact that males exhibited greater Total Displacement and Mean Velocity overall suggests that sex-specific baseline differences in postural behavior may be more robust than the direct effect of visual condition itself for certain variables. Third, the interaction observed for CoP Area underscores the importance of avoiding overly simple interpretations such as “visual occlusion is better” or “darkness is worse.” Instead, a more appropriate interpretation is that altered visual environments may elicit different sensorimotor solutions in different subgroups, even when the average condition effect is null. This interpretation is consistent with evidence indicating that postural responses under altered sensory conditions are dynamic and context-dependent and that task-related increases in sway are not expressed uniformly across all groups [4,13].

From a clinical and applied perspective, these findings support a more individualized approach to balance assessment and training. Protocols that manipulate visual input—whether through low-light exposure, blindfolding, or intermittent visual occlusion—may still be useful, but their effects should not be assumed to be uniform across participants or interchangeable across visual conditions. In practical terms, eyes-open-in-darkness conditions may be considered when the aim is to expose individuals to ambiguous or unreliable visual information, whereas complete visual occlusion may be considered when the goal is to reduce visual dependence and encourage reliance on non-visual sensory cues. Accordingly, an individualized selection of reduced-vision paradigms may be appropriate depending on the specific goal of assessment or training. Eyes-open-in-darkness paradigms may be more appropriate when the aim is to challenge sensory integration under conditions of limited or unreliable visual input, whereas complete visual occlusion paradigms may be preferable when the goal is to facilitate greater reliance on proprioceptive and vestibular information during balance training. However, these potential applications should be interpreted cautiously, as the present study examined immediate postural responses rather than long-term training adaptations. Previous research has shown that visual occlusion paradigms can enhance balance training outcomes in specific intervention settings [9,10,11,12]. The present findings do not contradict this literature, but they suggest that the acute postural response to altered visual input in a static task may depend on participant characteristics and the specific outcome measure being analyzed. Therefore, clinicians and researchers should interpret sway metrics with attention to variable-specific and subgroup-specific patterns rather than relying solely on global assumptions about vision loss. Such an approach may help refine the selection of sensory-challenging strategies according to the purpose of assessment or training, while avoiding the assumption that all reduced-vision paradigms impose equivalent demands on postural control.

The present study has several methodological strengths. First, a within-subject experimental design was employed, allowing each participant to serve as their own control and thereby reducing inter-individual variability in the comparison between visual conditions. Second, visual conditions were carefully standardized, with illumination levels maintained below 1 lux, ensuring a clear and reproducible distinction between degraded visual input and complete visual occlusion. Third, postural sway was assessed using a force platform, providing objective quantification of multiple CoP-derived outcomes, which are widely used as valid indicators of postural control [29,31]. Finally, the use of single-leg stance increased the sensitivity of the protocol relative to simpler bipedal tasks and provided a more demanding test of balance under altered sensory conditions [1].

Several limitations should also be acknowledged. First, the sample consisted exclusively of healthy young adults, which limits the generalizability of the findings to older adults, clinical populations, or individuals with pronounced vestibular or neurological impairment, who may rely on different sensory strategies. Second, visual status was assessed on the basis of mild visual deficits, as recorded from participants’ reported visual history and corrective lens prescription where applicable, rather than objective ophthalmologic or optometric evaluation. As a result, the subgroup with mild visual deficits may have been heterogeneous, and the severity or functional relevance of those deficits could not be precisely quantified. Third, although some variables still exhibited substantial variability, especially in measures such as CoP Area, this may have reduced the sensitivity to detect smaller effects. In addition, the sample size, while adequate for the analyses performed, may not have been sufficient to detect subtle subgroup differences, particularly in the mild-visual-deficit comparisons. Finally, the repeated-measures design of the present study allows conclusions about immediate postural responses under two visual conditions, but not about longer-term adaptation or training effects. Future studies using longitudinal or intervention-based designs may help clarify whether repeated exposure to degraded and occluded visual environments leads to stable sensorimotor adaptations over time.

## 5. Conclusions

In conclusion, the present study demonstrated that altered visual input does not exert a uniform or universally destabilizing effect on postural control during single-leg stance in healthy young adults. Although no overall main effect of visual condition was observed, the significant interaction between visual condition and sex for CoP Area indicates that males and females may adapt differently to degraded versus absent visual information. Moreover, males exhibited greater Total Displacement and Mean Velocity overall, suggesting sex-related differences in sway behavior independent of visual condition. These sex-related findings remained significant after adjustment for body weight, indicating that they were not explained by body weight. The absence of significant differences according to mild visual deficits further suggests that mild visual deficits may be effectively compensated for in this population under controlled balance conditions. Taken together, these findings support a more differentiated view of postural regulation under altered visual environments, emphasizing that the effects of visual manipulation depend not only on sensory context but also on individual characteristics. Importantly, the absence of a uniform effect of visual condition suggests that postural control in healthy young adults may remain robust under different forms of reduced visual input, likely due to effective multisensory compensation. This perspective may be particularly relevant for future research and the design of more targeted balance assessment and rehabilitation strategies.

## Figures and Tables

**Table 1:** Descriptive characteristics of the study participants according to sex. Continuous variables are presented as mean ± standard deviation, whereas categorical variables are presented as frequency (percentage). Between-group comparisons were performed using independent-samples *t*-tests for continuous variables and chi-square or Fisher’s exact tests for categorical variables.

Variable	Male (*n* = 21)	Female (*n* = 21)	*p*-Value (Between Groups)
Age (years), mean ± SD	20.71 ± 0.46	20.62 ± 0.50	0.52
Height (cm), mean ± SD	179.90 ± 5.68	166.86 ± 6.16	<0.001
Body weight (kg), mean ± SD	79.67 ± 11.37	63.14 ± 6.11	<0.001
BMI (kg/m^2^), mean ± SD	24.62 ± 3.78	22.95 ± 2.01	0.08
Dominant limb—Right, *n* (%)	20 (95.2)	18 (85.7)	0.60
Dominant limb—Left, *n* (%)	1 (4.8)	3 (14.3)	
Mild visual deficits—Yes, *n* (%)	8 (38.1)	9 (42.9)	0.75
Mild visual deficits—No, *n* (%)	13 (61.9)	12 (57.1)	

**Table 2:** Descriptive statistics and mixed ANOVA results for postural sway variables according to visual condition and sex.

Variable	Condition	Male (Mean ± SD)	Female (Mean ± SD)	Total (Mean ± SD)	F Condition Effect	*p*	*η*^2^p	F Condition × Sex Interaction	*p*	*η*^2^p	F Sex Effect	*p*	*η*^2^p
CoP Area (mm^2^)	EO	3068.54 ± 1155.61	3265.69 ± 1861.03	3167.12 ± 1533.25	0.014	0.906	0.000	9.910	0.003	0.199	1.634	0.208	0.039
	VO	3730.44 ± 1435.73	2551.69 ± 1156.11	3141.07 ± 1418.93									
Total Displacement (mm)	EO	2074.51 ± 521.35	1715.97 ± 573.63	1895.24 ± 570.99	0.446	0.508	0.011	0.406	0.528	0.010	9.212	0.004	0.187
	VO	2179.75 ± 462.94	1718.44 ± 473.26	1949.10 ± 517.97									
Mean Velocity (mm/s)	EO	103.34 ± 25.98	85.78 ± 28.13	94.56 ± 28.18	0.411	0.525	0.010	0.481	0.492	0.012	9.090	0.004	0.185
	VO	108.59 ± 23.06	85.57 ± 23.54	97.08 ± 25.80									
Oscillation Width (mm)	EO	45.09 ± 5.75	44.37 ± 8.26	44.73 ± 7.04	1.373	0.248	0.033	0.585	0.449	0.014	1.197	0.280	0.029
	VO	47.79 ± 5.78	44.94 ± 7.67	46.36 ± 6.86									
Oscillation Height (mm)	EO	102.58 ± 41.63	98.27 ± 44.90	100.43 ± 42.82	0.000	0.993	0.000	1.091	0.303	0.027	1.348	0.253	0.033
	VO	109.28 ± 28.55	91.46 ± 31.72	100.37 ± 31.14									

Values are presented as mean ± standard deviation (SD). EO = eyes open in darkness; VO = complete visual occlusion. The “Condition effect” refers to the main effect of visual condition across all participants, whereas the “Condition × Sex interaction” refers to whether the effect of visual condition differed between males and females. Higher values indicate greater postural sway.

**Table 3:** Independent samples t-test results according to mild visual deficits.

Variable	Condition	Mild Visual Deficits	N	Mean ± SD	*t*(df)	*p*
CoP Area (mm^2^)	EO	Yes	17	3464.29 ± 1850.09	1.037 (40)	0.306
		No	25	2965.03 ± 1276.35		
	VO	Yes	17	3279.41 ± 1496.88	0.516 (40)	0.608
		No	25	3047.00 ± 1386.70		
Total Displacement (mm)	EO	Yes	17	1970.82 ± 665.46	0.703 (40)	0.486
		No	25	1843.85 ± 504.92		
	VO	Yes	17	1939.37 ± 450.11	−0.099 (40)	0.922
		No	25	1955.71 ± 568.47		
Mean Velocity (mm/s)	EO	Yes	17	98.53 ± 32.49	0.750 (40)	0.458
		No	25	91.85 ± 25.19		
	VO	Yes	17	96.64 ± 22.47	−0.091 (40)	0.928
		No	25	97.39 ± 28.28		
Oscillation Width (mm)	EO	Yes	17	44.04 ± 8.18	−0.524 (40)	0.603
		No	25	45.21 ± 6.28		
	VO	Yes	17	46.94 ± 7.55	0.449 (40)	0.656
		No	25	45.97 ± 6.48		
Oscillation Height (mm)	EO	Yes	17	114.33 ± 50.04	1.781 (40)	0.083
		No	25	90.97 ± 35.12		
	VO	Yes	17	104.88 ± 37.22	0.770 (40)	0.446
		No	25	97.30 ± 26.62		

EO = eyes open in darkness; VO = complete visual occlusion.

## Data Availability

The data that support the findings of this study are openly available in Zenodo at https://doi.org/10.5281/zenodo.19327515 (accessed on 21 May 2026).
